# Impaired left atrial strain is associated with acute ischemic stroke in patients without atrial fibrillation

**DOI:** 10.3389/fstro.2026.1838151

**Published:** 2026-07-14

**Authors:** Robert Trueick, Jonathan Shpigelman, Omar Dabash, John McCallig, Muhammad Ahmad Ashfaque, Muneeb Quidwai, Lisa Donaghy, Patricia Guilfoyle, Lavanya Saiva, James O'Neill, Michael J. Daly

**Affiliations:** 1 Department of Cardiology, Connolly Hospital, Dublin, Ireland; 2 Department of Internal Medicine, Southern Illinois University School of Medicine, Springfield, IL, United States; 3 School of Medicine, Royal College of Surgeons in Ireland (RCSI) University of Medicine and Health Sciences, Dublin, Ireland

**Keywords:** acute ischemic stroke, cryptogenic stroke, ESUS, left atrial cardiomyopathy, left atrial strain, speckle-tracking echocardiography

## Abstract

**Background:**

Cryptogenic stroke, defined as a stroke with an unknown cause, poses a significant clinical challenge in cardiology and neurology. Left atrial (LA) dysfunction, as measured by left atrial strain (LAS), may identify patients who are at high risk for acute ischemic stroke (AIS), even if they do not have documented atrial fibrillation (AF).

**Methods:**

This retrospective, cross-sectional and cohort study (known as the ASSISTANT Study) was conducted at a single, tertiary-care referral center. The study included patients who tested positive on the Face Arm Speech Test (FAST) and who did not have high-risk stroke mechanisms. These patients presented acutely and underwent a transthoracic echocardiogram. Speckle-tracking echocardiography was used to measure LAS in three phases: reservoir (LASr), conduit (LAScd), and contractile (LASct). The associations of LAS with imaging-confirmed acute ischemic stroke (AIS) at presentation and incident AIS during follow-up were evaluated using multivariable logistic and Cox regression analyses, respectively.

**Results:**

Among the 415 patients (251 cases and 164 controls), LASr and LAScd were both independently associated with AIS at presentation [OR per 5% increase: 0.789 (95% CI, 0.674–0.920) and 0.573 (95% CI, 0.450–0.718), respectively]. During a median follow-up of 3.59 years, 31 patients developed incident AIS. The incidence of AIS increased as the tertiles of LASr (*P*_trend_ = 0.021) and LAScd (*P*_trend_ < 0.001) worsened. Only LAScd remained independently associated with incident AIS [HR per 5% increase: 0.682 (95% CI, 0.478–0.953)].

**Conclusion:**

Impaired LAScd is independently associated with prevalent and incident AIS in patients without identified high-risk stroke mechanisms. Prospective studies are needed to determine whether LAScd can guide risk stratification and preventive strategies in this patient population.

**Trial Registration:**

ClinicalTrials.gov (NCT07102693).

## Introduction

Every year, 12 million new strokes occur worldwide (Feigin et al., 2025), with ischemic strokes accounting for the vast majority. Even after extensive evaluations, including cardiac rhythm monitoring, carotid imaging, transthoracic echocardiogram (TTE), and, in selected cases, transesophageal echocardiography, up to one-third of ischemic strokes remain without a clear etiology (Marnane et al., 2010). In many patients, neuroimaging demonstrates an embolic-appearing infarct pattern, and these cases are therefore classified as embolic stroke of undetermined source (ESUS) (Hart et al., 2014). ESUS is a heterogeneous construct encompassing several potential embolic mechanisms, including non-stenotic extracranial or intracranial atherosclerotic plaques, prothrombotic states such as cancer-associated thrombosis, and right-to-left shunts (Ntaios et al., 2024). Despite receiving guideline-based secondary preventive therapy, the ESUS annual stroke recurrence rate is 4%−5% (Hart et al., 2017), which is significantly greater than that of non-ESUS strokes (Putaala et al., 2015; Ntaios et al., 2015).

Left atrial cardiomyopathy (LACM), which can occur even in the absence of atrial fibrillation (AF), has been proposed to contribute to these strokes (Kamel and Healey, 2017; Habibi et al., 2019). LACM is characterized by reduced left atrial (LA) myocyte contractility, endothelial dysfunction, and fibrosis (Darlington and McCauley, 2020). These factors may create a thromboembolic substrate independent of AF, although this concept remains a subject of debate. Interestingly, in the Cryptogenic Stroke and Underlying Atrial Fibrillation (CRYSTAL-AF) trial, 70% of patients with cryptogenic stroke did not develop AF after 3 years of continuous cardiac rhythm monitoring with an implantable loop recorder (ILR) (Sanna et al., 2014). Additionally, a temporal analysis of the ASSERT trial revealed that 31% of patients with both subclinical AF and ischemic stroke or systemic embolism only had the subclinical AF detected after the event, despite having undergone a median of 228.5 days of continuous monitoring beforehand (Brambatti et al., 2014). Similarly, a subgroup analysis of the TRENDS study, which utilized implantable devices for continuous rhythm monitoring, indicated that 73% of patients with cerebrovascular events or systemic embolisms had no detectable atrial tachyarrhythmia in the 30 days leading up to the event (Daoud et al., 2011). Importantly, although the implantation of an ILR after an ischemic stroke increased AF detection (13% vs. 2.4% at 12 months) and was associated with a higher initiation of oral anticoagulation, there was no significant reduction in recurrent stroke or transient ischemic attack (TIA) (Ko et al., 2022).

Throughout the cardiac cycle, the LA operates in three phases (Hoit, 2014). During ventricular systole, the reservoir phase allows filling of the LA from the pulmonary veins. The mitral valve opens at the onset of ventricular diastole, allowing passive filling of the left ventricle (LV) during the conduit phase. The final contractile “booster pump” phase occurs during atrial systole, augmenting LV filling. LA strain (LAS), measured using speckle-tracking echocardiography, enables semi-automated quantification of these three phases and can detect LA dysfunction earlier than LA volume (LAV) alone (Cameli et al., 2012). Small studies have shown an association between impaired LAS and the incidence of cryptogenic stroke in patients without a history of AF (Pagola et al., 2014).

Therefore, we aimed to further investigate the association between LAS and acute ischemic stroke (AIS) in patients without AF or other identifiable stroke mechanisms.

## Methods

### Study design and patient population

**Figure 1:**
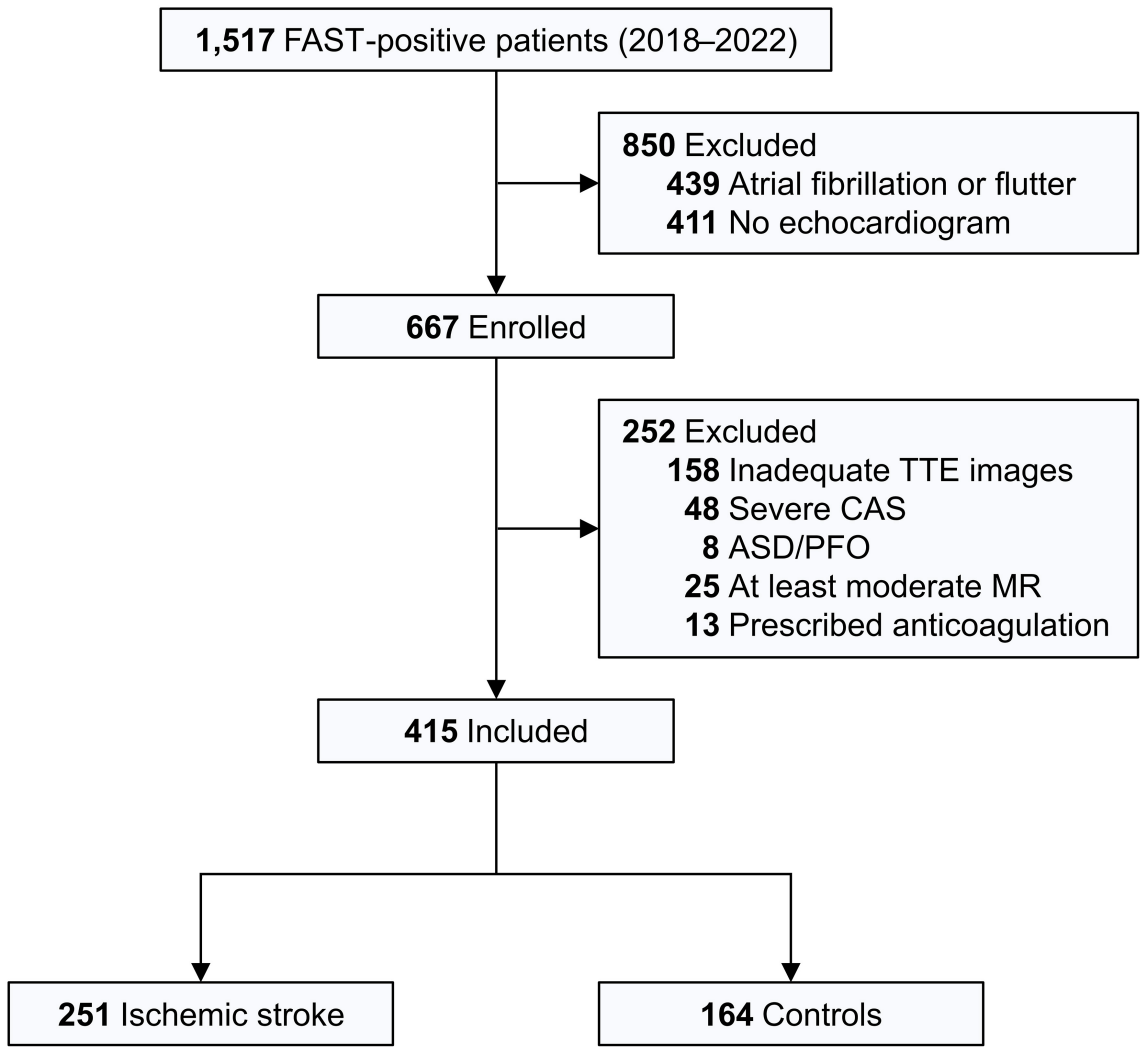
Flow diagram of patient selection. ASD, atrial septal defect; CAS, carotid artery stenosis; MR, mitral regurgitation; PFO, patent foramen ovale.

We conducted a single-center retrospective study utilizing both cross-sectional and cohort analyses. At Connolly Hospital in Blanchardstown, Dublin, Ireland, we identified patients aged ≥18 years who presented to the emergency department between January 2018 and December 2022 with a positive Face Arm Speech Test (FAST) (Nor et al., 2004) and underwent brain CT ± MRI and TTE as part of their diagnostic workup. Patients were excluded from the study if they met any of the following criteria: (i) documented AF or atrial flutter, (ii) TTE images of insufficient quality for LAS analysis, (iii) severe ipsilateral carotid artery stenosis, (iv) atrial septal defect or patent foramen ovale, (v) at least moderate mitral regurgitation, and/or (vi) prescription of anticoagulation medication (Figure 1).

### Echocardiographic assessment

**Figure 2:**
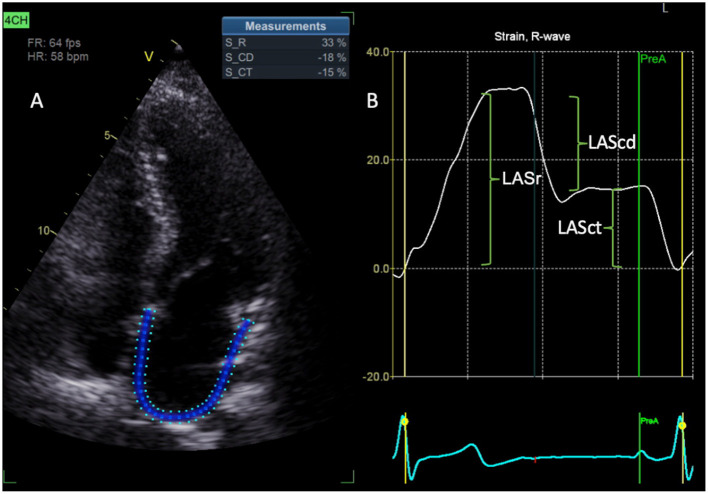
LAS assessment using 2D speckle-tracking echocardiography in a representative patient who presented with AIS. **(A)** Apical 4-chamber view with left atrial endocardial border tracings. **(B)** R-R gated LAS curve with annotations depicting the three phases of the LA cycle: LASr, LAScd, and LASct. AIS, acute ischemic stroke; LAS, left atrial strain; LAScd, left atrial conduit strain; LASct, left atrial contractile strain; LASr, left atrial reservoir strain.

Two doctors who were blinded to AIS outcomes retrospectively analyzed TTE images obtained at initial presentation using EchoPAC Software v204 (GE Vingmed Ultrasound AS, Horten, Norway). LAS was assessed by speckle-tracking echocardiography, with the LA endocardial borders traced automatically after the manual selection of landmark points in the apical 4-chamber (A4c) view at end-systole (Singh et al., 2019; Cameli et al., 2009). For all LAS measurements, R-R gating was used to set the zero-reference point. LA reservoir (LASr), conduit (LAScd), and contractile (LASct) strains were calculated as the peak longitudinal deformations of the LA during ventricular systole, early ventricular diastole, and atrial systole, respectively. Since LAScd and LASct are negative by convention, they were multiplied by −1 such that higher LAS values uniformly reflected better atrial function. A representative example of LAS assessment is shown in Figure 2. LAVs were measured automatically in the A4c view: maximum (LAVmax) at the end of ventricular systole, minimum (LAVmin) at the end of ventricular diastole, and pre-atrial contraction (LAVpreA) immediately before atrial systole. The left ventricular ejection fraction (LVEF) was calculated using the modified Simpson's biplane technique. The interventricular septal wall thickness at end-diastole (IVSd) and the E/e′ ratio were measured according to established international standards.

### Cross-sectional analysis

Patients with neuroimaging-confirmed AIS at index presentation were classified as cases, whereas the others were classified as controls. The associations of LAS and LAV parameters with AIS were examined using logistic regression and were reported as odds ratios (ORs) with 95% confidence intervals (CIs). Multivariable models were adjusted for an *a priori* set of covariates identified at index presentation, including age, sex, hypertension, dyslipidemia, diabetes mellitus, smoking status, prior AIS, LVEF, IVSd, and the E/e′ ratio.

### Cohort analysis

To identify incident AIS, we reviewed data from the National Integrated Medical Imaging System (NIMIS) in Ireland, focusing on each patient's index presentation until 31 December 2024. The median follow-up duration was estimated using the reverse Kaplan–Meier (KM) method (Schemper and Smith, 1996). Patients were right-censored either at the time of death or at the end of the follow-up period. The cumulative incidence (1 – KM) curves for AIS were stratified by tertiles of baseline LAS/LAV parameters and compared using standard and trend log-rank tests. Cox proportional hazards regression was used to estimate hazard ratios (HRs) with 95% CIs; the proportional hazards assumption was verified using the Grambsch–Therneau test of scaled Schoenfeld residuals. To preserve parsimony, given the limited number of incident events, multivariable models were adjusted for a reduced set of covariates (age, dyslipidemia, diabetes mellitus, and smoking status), which were selected for clinical relevance and supported by their univariable associations with AIS at index presentation. Additionally, newly diagnosed AF was identified within a standardized 2-year window after index presentation, based on a retrospective review of available clinical documents, which corresponds to the shortest available longitudinal follow-up in the cohort. Rhythm-monitoring strategies were not standardized and were performed at the treating physician's discretion. Between-group comparisons were used to evaluate differences in AF incidence between cases and controls, between patients who subsequently developed AIS and those who did not, and in baseline LAS based on the subsequent AF status of the patients.

### Statistical analysis

Continuous variables are presented as mean ± standard deviation (SD), and categorical variables are expressed as counts (percentage). Unpaired *t*-tests with Welch's correction for unequal variances were used to compare continuous data, whereas the chi-square test was used for categorical data. The E/e′ ratio was missing for 22% of participants; primary multivariable logistic regression models utilized complete-case analysis, and a sensitivity analysis excluding the E/e′ ratio from the adjustment set assessed the robustness of the results while maximizing the sample size. A two-tailed *P*-value of < 0.05 was considered statistically significant. All analyses were conducted using R version 4.5 (R Foundation for Statistical Computing, Vienna, Austria).

## Results

### Characteristics of the study population

The baseline characteristics of the study cohort are summarized in Table 1. The study included 415 patients (56.4% men; 64.6 ± 14.9 years), with 251 (60.5%) having neuroimaging-confirmed AIS. Patients with AIS were older (65.9 vs. 62.7 years, *P* = 0.048) and had higher rates of dyslipidemia (39.4% vs. 29.9%, *P* = 0.047), diabetes mellitus (24.7% vs. 16.5%, *P* = 0.046), and current smoking (23.9% vs. 15.9%, *P* = 0.048). With respect to echocardiographic parameters, LVEF was similar between groups (57.1% vs. 57.8%, *P* = 0.418), but AIS cases had worse LA indices: LASr (26.7% vs. 30.1%, *P* < 0.001), LAScd (11.3% vs. 15.1%, *P* < 0.001), LA emptying fraction (LAEF) [57.3% vs. 60.0%, *P* = 0.009], LAVmin (18.8 mL vs. 16.4 mL, *P* = 0.009), and LAVpreA (33.4 mL vs. 29.2 mL, *P* = 0.005).

**Table 1:** Baseline characteristics of the study population.

Variable	Total (*N* = 415)	Cases (*n* = 251)	Controls (*n* = 164)	*P*-value
Clinical characteristics	
Age, years	64.6 ± 14.9	65.9 ± 13.0	62.7 ± 17.4	0.048
Male patients	234 (56.4)	143 (57.0)	91 (55.5)	0.766
Prior AIS	16 (3.9)	10 (4.0)	6 (3.7)	0.866
Hypertension	251 (60.5)	157 (62.5)	94 (57.3)	0.286
Dyslipidemia	148 (35.7)	99 (39.4)	49 (29.9)	0.047
Diabetes mellitus	89 (21.4)	62 (24.7)	27 (16.5)	0.046
Current smoker	86 (20.7)	60 (23.9)	26 (15.9)	0.048
Family history of CVD	19 (4.6)	8 (3.2)	11 (6.7)	0.093
OSA	30 (7.2)	22 (8.8)	8 (4.9)	0.135
Echocardiography	
LASr, %	28.1 ± 8.7	26.7 ± 9.0	30.1 ± 8.0	< 0.001
LAScd, %	12.8 ± 6.7	11.3 ± 6.1	15.1 ± 6.9	< 0.001
LASct, %	15.3 ± 6.1	15.4 ± 6.8	15.0 ± 4.9	0.531
LAEF, %	58.4 ± 10.2	57.3 ± 10.3	60.0 ± 9.8	0.009
LAEV, mL	24.3 ± 10.4	24.1 ± 9.8	24.6 ± 11.2	0.619
LAVmin, mL	17.8 ± 9.9	18.8 ± 10.8	16.4 ± 8.2	0.009
LAVmax, mL	42.1 ± 18.0	42.8 ± 18.4	41.0 ± 17.4	0.313
LAVpreA, mL	31.7 ± 15.1	33.4 ± 15.7	29.2 ± 13.8	0.005
IVSd, cm	1.0 ± 0.3	1.0 ± 0.2	1.0 ± 0.3	0.223
E/e′^a^	11.3 ± 4.4	11.4 ± 4.7	11.0 ± 3.9	0.370
LVEF, %	57.4 ± 8.9	57.1 ± 9.5	57.8 ± 8.0	0.418

Continuous variables are presented as the mean ± SD and categorical variables are presented as counts (percentage). AIS, acute ischemic stroke; CVD, cardiovascular disease; IVSd, interventricular septal thickness at end-diastole; LAEF, left atrial emptying fraction; LAEV, left atrial emptying volume; LAScd, left atrial conduit strain; LASct, left atrial contractile strain; LASr, left atrial reservoir strain; LAVmax, left atrial maximum volume; LAVmin, left atrial minimum volume; LAVpreA, left atrial pre-contraction volume; LVEF, left ventricular ejection fraction; OSA, obstructive sleep apnea. ^a^Based on 324 patients (191 cases, 133 controls).

### Associations between LA function and AIS at index presentation

**Figure 3:**
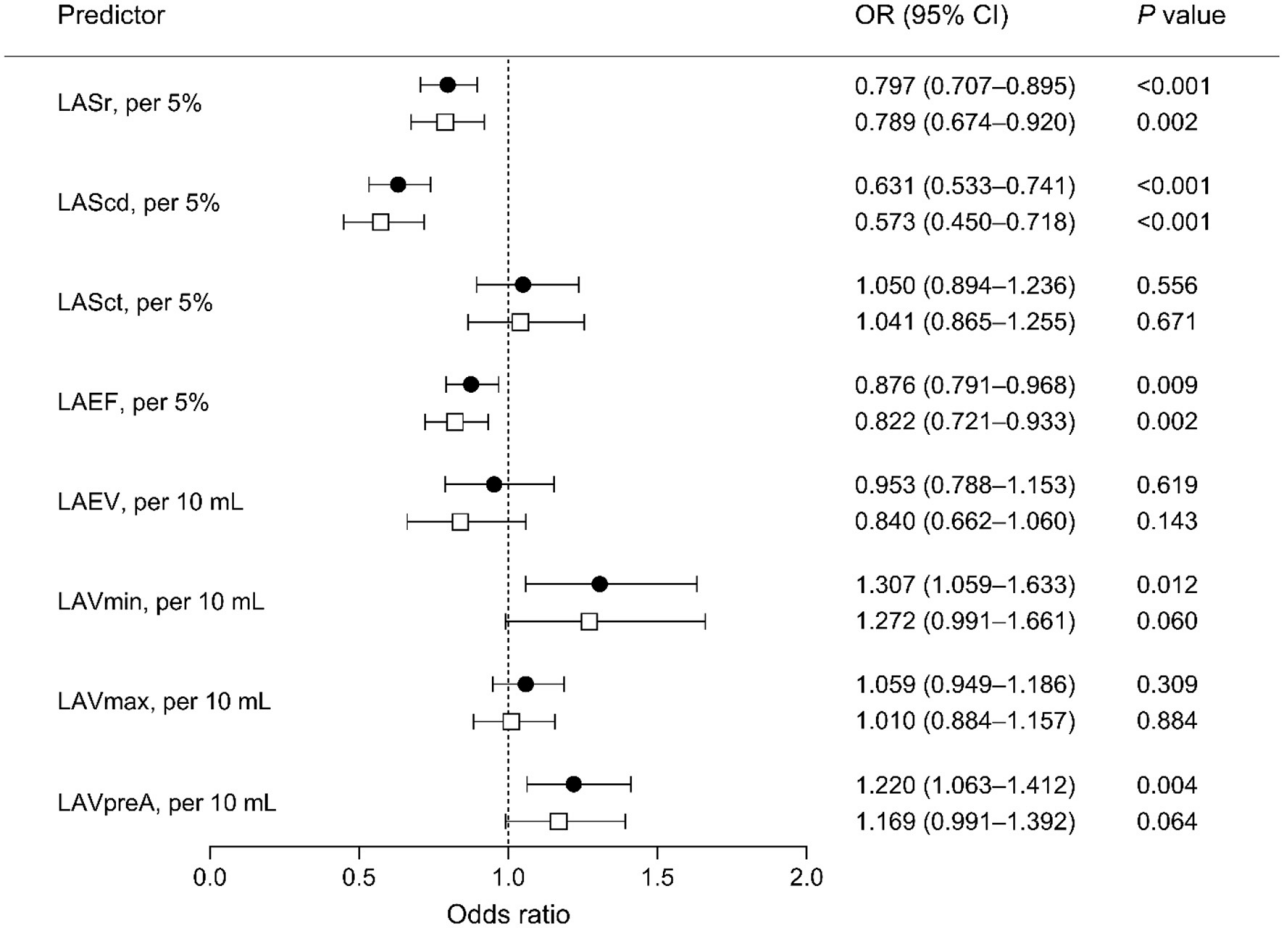
Associations of LAS and LAV with AIS at index presentation. The solid circles and open squares represent the ORs for univariable and multivariable models, respectively; the whiskers represent the 95% CIs. AIS, acute ischemic stroke; CI, confidence interval; LAEF, left atrial emptying fraction; LAEV, left atrial emptying volume; LAS, left atrial strain; LAScd, left atrial conduit strain; LASct, left atrial contractile strain; LASr, left atrial reservoir strain; LAVmax, left atrial maximum volume; LAVmin, left atrial minimum volume; LAVpreA, left atrial pre-contraction volume; OR, odds ratio.

Figure 3 summarizes the associations between LAS/LAV parameters and AIS at index presentation. In univariable analysis, LASr, LAScd, LAEF, LAVmin, and LAVpreA were associated with AIS, with the strongest associations observed for LASr [OR per 5% increase, 0.797 (95% CI, 0.707–0.895)] and LAScd [OR per 5% increase, 0.631 (95% CI, 0.533–0.741)]. After adjusting for covariates, only LASr [OR per 5% increase, 0.789 (95% CI, 0.674–0.920)], LAScd [OR per 5% increase, 0.573 (95% CI, 0.450–0.718)], and LAEF [OR per 5% increase, 0.822 (95% CI, 0.721–0.933)] remained independently associated with AIS. Sensitivity analyses that excluded the E/e′ ratio from the adjustment set yielded directionally consistent effect estimates with similar magnitudes (Supplementary Table S1).

Since LAScd demonstrated the strongest and most consistent association with AIS, we performed an exploratory analysis to assess its incremental value. The addition of LAScd to the covariate-only model increased the C-statistic from 0.607 (95% CI, 0.544–0.671) to 0.693 (95% CI, 0.634–0.752), with a significant improvement in model fit (likelihood ratio test *P* < 0.001).

### Associations between LA function and future AIS

**Figure 4:**
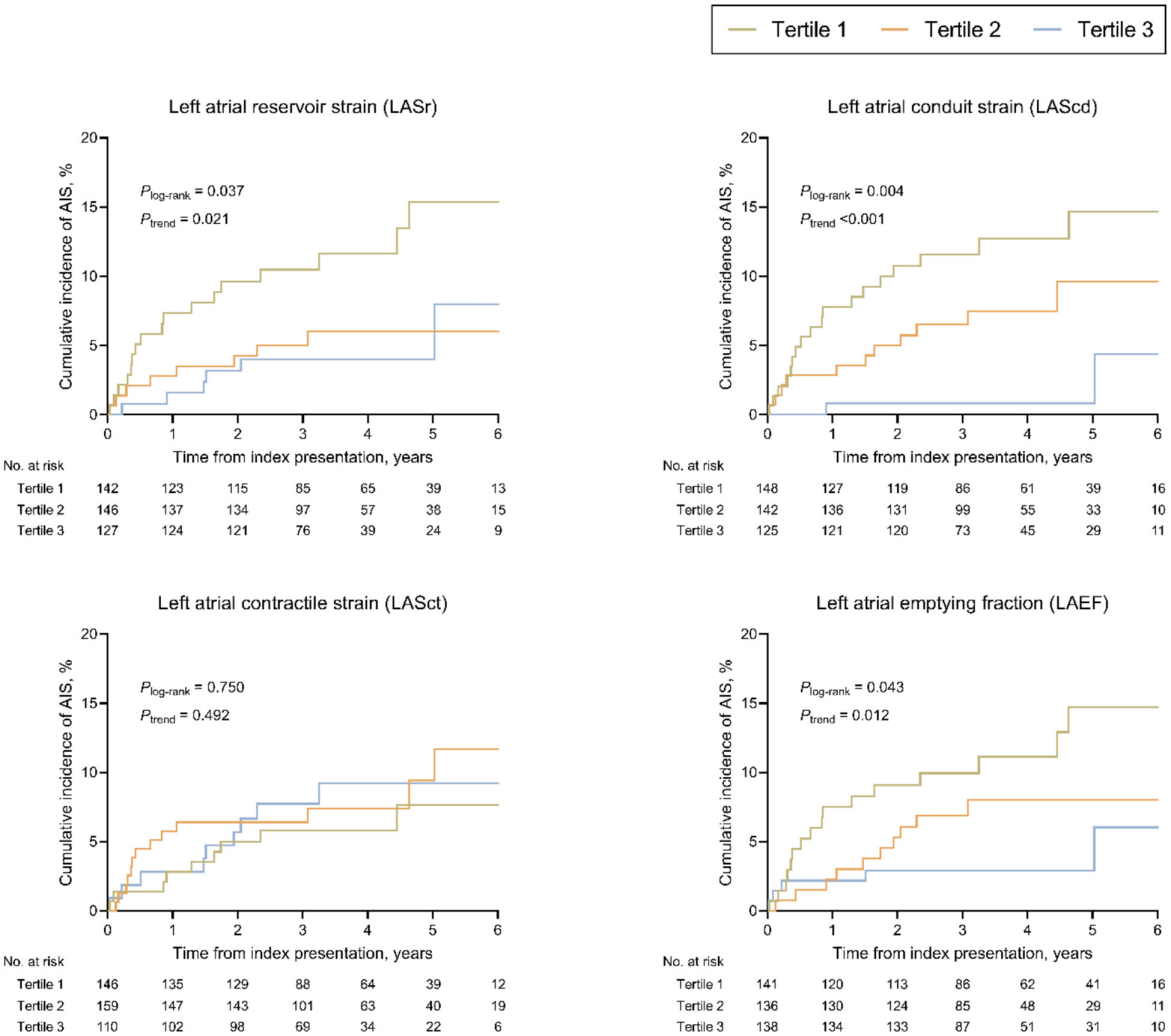
Cumulative incidence (1 – KM) curves for future AIS stratified by tertiles of baseline LAS or LAEF. Patients were right-censored at death or at the end of the follow-up period. The tertile cut points were as follows: LASr (24.0%, 32.0%), LAScd (9%, 15%), LASct (12%, 18%), and LAEF (54%, 63%). AIS, acute ischemic stroke; KM, Kaplan–Meier; LAEF, left atrial emptying fraction; LAS, left atrial strain; LAScd, left atrial conduit strain; LASct, left atrial contractile strain; LASr, left atrial reservoir strain.

Over a median follow-up of 3.59 years (95% CI, 3.33–3.81), 27 patients (6.5%) died before developing AIS, and 31 patients (7.5%) experienced an incident AIS. Of these, 27 (87.1%) were already prescribed antiplatelet therapy. Figure 4 depicts the cumulative incidence (1 – KM) of future AIS, stratified by tertiles of baseline LAS and LAEF. The incidence differed across tertiles for LASr (*P*_log − rank_ = 0.037), LAScd (*P*_log − rank_ = 0.004), and LAEF (*P*_log − rank_ = 0.043). Furthermore, all three exhibited significant trends, with the trend for LAScd tertiles being the most prominent (*P*_trend_ < 0.001). A stepwise increase in the cumulative incidence of AIS across worsening LAScd tertiles was observed, approaching 15% by 5 years among patients with the greatest impairment in LAScd (Tertile 1). In univariable Cox regression analysis, LASr, LAScd, and LAEF were each associated with incident AIS; however, only LAScd remained independently associated with incident AIS after adjusting for covariates [HR per 5% increase, 0.682 (95% CI, 0.478–0.953); Table 2].

**Table 2:** Associations of LAS and LAEF with incident AIS.

Predictor	Univariable model		Multivariable model	
	HR (95% CI)	*P*-value	HR (95% CI)	*P*-value
LASr, per 5%	0.788 (0.631–0.973)	0.026	0.845 (0.668–1.057)	0.142
LAScd, per 5%	0.623 (0.445–0.850)	0.002	0.682 (0.478–0.953)	0.024
LASct, per 5%	1.010 (0.753–1.330)	0.944	1.024 (0.763–1.355)	0.870
LAEF, per 5%	0.823 (0.702–0.971)	0.021	0.865 (0.732–1.026)	0.096

AIS, acute ischemic stroke; LAEF, left atrial emptying fraction; LAScd, left atrial conduit strain; LASct, left atrial contractile strain; LASr, left atrial reservoir strain.

### Newly diagnosed AF during follow-up

Within 2 years of index presentation, AF was diagnosed in 18 patients (4.3%). There was no difference in incidence between cases and controls (3.6% vs. 5.5%; *P* = 0.460) or between patients who later developed AIS and those who did not (6.5% vs. 4.2%; *P* = 0.636). Similarly, LASr (25.9% vs. 28.2%; *P* = 0.290), LAScd (11.6% vs. 12.8%; *P* = 0.448), and LASct (14.6% vs. 15.3%; *P* = 0.615) did not differ significantly between patients with and without AF.

## Discussion

In this study, we investigated the association between LA function and AIS in 415 patients without AF or other conventional cardioembolic or large-artery sources of stroke. On initial comparison, there was no difference in LV function between patients with and without AIS. However, several indices of LA function—including LASr, LAScd, LAEF, LAVmin, and LAVpreA—were significantly worse in subjects with AIS. After adjusting for known stroke risk factors and LV systolic and diastolic function, only LASr, LAScd, and LAEF were independently associated with AIS at index presentation. Among these indices, LAScd showed the strongest association; when expressed conversely, each 5% reduction in LAScd corresponded to a 74.5% higher odds of AIS at presentation (Figure 3).

Similarly, our cohort study showed that AIS incidence increased across worsening LASr, LAScd, and LAEF tertiles. LAScd again exhibited the strongest association, with wide separation between tertile-specific incidence curves at all time points (Figure 4). Furthermore, in Cox regression, LAScd was the only parameter independently associated with incident AIS. When considering the inverse association, each 5% decrease in LAScd was associated with a 46.6% higher hazard of incident AIS (Table 2). Although acute stroke can transiently affect cardiac function (Scheitz et al., 2022), the concordant cross-sectional and longitudinal findings from TTEs at index presentation, together with the lack of LVEF differences between cases and controls, suggest that impaired LAS is unlikely to be entirely explained by acute cerebral injury. However, since the timing of echocardiography in relation to stroke onset was not recorded, we cannot exclude the possibility of neurocardiac effects on LAS measurements in some patients.

Despite prior evidence linking maximal LA volume to cerebrovascular events (Tsang et al., 2006), we found no association between LA volume and AIS after adjusting for covariates. This suggests that structural indices may not adequately capture the cardioembolic stroke risk associated with LA dysfunction. Instead, our results align with the growing body of evidence indicating that impaired LAS is associated with an elevated risk of ischemic stroke (Mannina et al., 2023), which may have important clinical implications. In ESUS, where antiplatelet therapy remains the standard for secondary prevention (Ford et al., 2022), impaired LAS may help identify a subgroup in whom anticoagulation warrants prospective evaluation. Indeed, the majority of future AIS events in our study occurred in patients already receiving antiplatelet therapy.

Although the relationship between LACM and cardioembolism remains incompletely understood, prior studies have shown that left atrial blood stasis, assessed by spontaneous echo contrast, can occur in normal sinus rhythm and is associated with depressed atrial function, LA thrombus, and cerebrovascular events (Sadanandan and Sherrid, 2000). In this context, our finding that LAScd exhibited the strongest association with AIS is notable, as LAScd reflects passive LA emptying during early diastole and generally accounts for the majority of LV filling (Thomas et al., 2019).

One common hypothesis is that impaired LAS reflects undiagnosed paroxysmal AF (Pagola et al., 2014). In our cohort, all patients were in sinus rhythm at the time of imaging, and rates of subsequent AF diagnosis did not differ by case-control status at index presentation or by whether patients later developed AIS. Similarly, baseline LAS did not differ between patients who did and did not develop AF during follow-up. However, rhythm-monitoring strategies were not standardized or systematically recorded, and subclinical AF may therefore have gone undetected. Accordingly, we cannot exclude the possibility that impaired LAS identifies, at least in some patients, an atrial substrate associated with undiagnosed paroxysmal AF rather than an independent manifestation of LACM. Even if impaired LAS reflects a subclinical atrial substrate that predisposes patients to AF, it may provide additional sensitivity for identifying cardioembolic risk beyond rhythm monitoring alone. LAS has the advantage of being readily incorporated into the evaluation of cryptogenic stroke patients. It is measured in a time- and cost-efficient manner using existing data available from standard diagnostic echocardiograms, requiring no additional testing.

Previous randomized trials of anticoagulation in patients with ESUS have failed to demonstrate benefit (Diener et al., 2019; Hart et al., 2018; Kamel et al., 2024). ARCADIA was the only trial to specifically include patients with LACM; however, its only echocardiographic criterion was an indexed LA diameter of ≥3 cm/m^2^, a structural measure that has since been superseded by more sensitive measurements of LACM, particularly LAS. For instance, in transthyretin amyloid cardiomyopathy, impaired LAS has been shown to predict thrombotic events independent of AF and without correlation to increased LA volumes (Ozbay et al., 2025).

Taken together, our findings demonstrate that impairment of the LA reservoir, and especially conduit function, is strongly associated with AIS. Prospective trials are therefore warranted to determine whether patients with impaired LAS and ESUS would benefit from anticoagulation to reduce recurrent stroke risk.

### Limitations

This study has several limitations. First, this was a single-center retrospective study, which may limit generalizability and preclude causal inference. The use of FAST-positive patients without AIS as controls reflected a heterogeneous real-world cohort undergoing standardized stroke evaluation, but this approach may introduce selection bias, as this group likely included both cerebrovascular syndromes without imaging-confirmed infarction and non-cerebrovascular stroke mimics. Final neurological diagnoses for control patients were not consistently available from retrospective chart review. Furthermore, although established high-risk stroke mechanisms were excluded, non-stenotic plaque vulnerability features, such as intraplaque hemorrhage or a thin/ruptured fibrous cap, were not systematically assessed.

Although the study cohort included 415 patients, only 31 experienced an incident AIS over a median follow-up of 3.59 years, reducing the statistical power of the cohort analysis relative to the cross-sectional analysis and potentially limiting model stability and the precision of estimates. The relatively young mean age of the cohort may also limit generalizability to older stroke populations, in whom AF, atrial remodeling, and recurrent ischemic events may be more prevalent. Outcome ascertainment in the cohort analysis relied on NIMIS, which should capture nearly all imaging-confirmed AIS nationwide; however, emigration could not be accounted for and may have led to a slight underestimation of stroke incidence.

Rather than using the preferred biplane method, left atrial strain was assessed from the A4c view alone, which is considered acceptable by the EACVI/ASE Task Force (Badano et al., 2018). LAS was measured using semi-automated software by clinicians blinded to the study outcomes; however, inter- and intra-observer variability were not assessed. Furthermore, although AF was diagnosed in only 4.3% of patients within 2 years, rhythm-monitoring strategies were not standardized, and details were not uniformly available upon retrospective review; hence, undetected paroxysmal AF cannot be excluded.

Finally, the timing of echocardiography in relation to stroke onset was not recorded, so acute neurocardiac effects on LAS measurements cannot be excluded.

## Summary

In this study, we explored the relationship between LA function and AIS in FAST-positive patients without AF or other identified high-risk stroke mechanisms. LASr and LAScd were independently associated with AIS at presentation, and incident AIS increased across worsening tertiles of both baseline LASr and LAScd during follow-up. However, after multivariable adjustment, only LAScd remained independently associated with incident AIS. Prospective studies are needed to determine whether LACM, particularly impaired LAScd, can guide risk stratification and preventive strategies in this population.

## Data Availability

The original contributions presented in the study are included in the article/Supplementary material, further inquiries can be directed to the corresponding author.
