# Supplementary material for: Impaired left atrial strain is associated with acute ischemic stroke in patients without atrial fibrillation
Source: Front Stroke. 2026 Jul 14;5:1838151. doi: 10.3389/fstro.2026.1838151 (PMC13407361; doi:10.3389/fstro.2026.1838151)
Supplement: Supplementary file 1 [file Table_1.docx]

**Supplementary Table 1.** Associations of LAS and LAV with AIS at index presentation: primary and sensitivity models according to adjustment for E/e’.

| Predictor | Primary model^a^ (N = 324)  OR (95% CI) | Sensitivity model^b^ (N = 415)  OR (95% CI) |
| --- | --- | --- |
| LASr, per 5% | 0.789 (0.674–0.920) | 0.788 (0.688–0.900) |
| LAScd, per 5% | 0.573 (0.450–0.718) | 0.588 (0.480–0.714) |
| LASct, per 5% | 1.041 (0.865–1.255) | 1.029 (0.871–1.219) |
| LAEF, per 5% | 0.822 (0.721–0.933) | 0.882 (0.791–0.981) |
| LAEV, per 10 mL | 0.840 (0.662–1.060) | 0.947 (0.770–1.163) |
| LAVmin, per 10 mL | 1.272 (0.991–1.661) | 1.302 (1.040–1.652) |
| LAVmax, per 10 mL | 1.010 (0.884–1.157) | 1.058 (0.939–1.195) |
| LAVpreA, per 10 mL | 1.169 (0.991–1.392) | 1.222 (1.051–1.431) |

^a^Adjusted for the prespecified covariates (complete-case sample)

^b^Adjusted for the prespecified covariates, excluding E/e’

AIS, acute ischemic stroke; CI, confidence interval; LAEF, left atrial emptying fraction; LAEV, left atrial emptying volume; LAS, left atrial strain; LAScd, left atrial conduit strain; LASct, left atrial contractile strain; LASr, left atrial reservoir strain; LAVmax, left atrial maximum volume; LAVmin, left atrial minimum volume; LAVpreA, left atrial pre-contraction volume; OR, odds ratio.
